# Navigating Sensitive Conversations: Patient-Centered Communication and Politeness Markers in Chinese Online Medical Consultations

**DOI:** 10.3390/healthcare12232465

**Published:** 2024-12-06

**Authors:** Yidi Wang, Xiaoya Yang, Jiaying Liu

**Affiliations:** 1 Department of Communication, University of California Santa Barbara, Santa Barbara, CA 93106, USA; yidiwang744@ucsb.edu; 2 School of Journalism and Communication, Wuhan University, Wuhan 430072, China; xiaoyayang@whu.edu.cn

**Keywords:** politeness markers, patient-centered communication, online medical consultation

## Abstract

**Background/Objectives**: In China, discussing sexual and reproductive health remains taboo, often preventing patients from seeking care or advice on sensitive topics. Online medical consultations (OMCs) offer a unique platform for patients to discuss these concerns more openly. This study investigates how patient-centered communication (PCC) practices, including conversational themes and the use of politeness markers, influence patient satisfaction in Chinese OMCs, with a focus on sensitive gynecology and andrology topics. **Methods**: This study used a mixed-methods approach, including theme-oriented discourse analysis (TODA) and content analysis on 328 OMCs (179 in andrology, 149 in gynecology) collected from Dr. Chunyu, a popular Chinese online healthcare platform that provides medical consultations, from 19 to 22 March 2022. Logistic regressions were conducted to assess the influence of politeness markers on patient satisfaction, while TODA examined PCC practices in sensitive conversations. **Results**: TODA identified two key themes in PCC that enhanced patient satisfaction: normalizing sensitive health concerns and fostering collaborative decision-making. Politeness markers, specifically the use of polite words and expressions of best wishes, were positively associated with patient satisfaction. However, downtoners, emojis, and sentence-final particles showed no significant effect. There were no significant differences in the impact of politeness markers between gynecology and andrology consultations. **Conclusions**: This study highlights the importance of PCC and politeness markers in improving patient satisfaction in OMCs, especially when addressing sensitive sexual health topics.

## 1. Introduction

In China, sex education has historically been neglected, with conservative coverage of sexual and reproductive health issues common in school curricula [1]. This deficiency has left many adolescents and young adults without the essential knowledge and guidance needed in these areas [2]. Chinese culture, which strongly emphasizes collectivist values like social stability and family harmony, tends to view individual expressions of sexuality, such as casual sex, as disruptive to these ideals. As a result, traditional views often frame sexuality in a restrictive manner [1]. This phenomenon is particularly pronounced in China, where cultural values and social norms around sexuality create stronger taboos compared to more liberal or individualistic societies [3,4,5]. Thus, sex-related topics are often viewed as taboo and private, leading many to feel discomfort or stigma when discussing sexual concerns or questions in public [6]. This cultural sensitivity extends to medical settings, making it challenging for patients to openly discuss their sexual health concerns with gynecology or andrology doctors during in-person consultations [7].

Additionally, despite the significant growth in the number of doctors in China over the past decade, the healthcare system continues to grapple with major regional disparities in allocation, particularly in the western regions, where shortages persist in both population coverage and geographic distribution [8]. These disparities contribute to strained offline doctor–patient relationships and a crisis of trust [9], further diminishing the likelihood of addressing sensitive topics like sexual education during face-to-face healthcare visits.

Online medical consultation (OMC) platforms provide an alternative avenue for patients to seek help and advice. Platforms like Dr. Chunyu, 120ask.com, Ali Medical Valley, and GoodDF.com facilitate consultations across a wide range of specialties, allowing patients to consult with doctors without the barriers of insurance coverage or geographic constraints. OMC platforms offer a convenient way for patients to access professional medical advice and support, particularly for managing their sexual and reproductive health. The anonymity provided by these platforms encourages individuals to discuss sensitive topics that might cause shame or embarrassment, such as sexually transmitted infections [10]. Although previous research has shown that tension in doctor–patient relationships can be alleviated when discussing general health issues in OMCs with trustworthy doctors [9], there is limited empirical research on how these dynamics unfold in OMCs addressing sexual and reproductive health concerns and their impact on patient satisfaction.

OMC platforms operate across diverse models based on communication modes (e.g., synchronous vs. asynchronous; text-only vs. video, audio, or hybrid), provider integration (e.g., integrated health systems with hospital and laboratory services vs. marketplace models connecting patients with independent providers), payment structures (e.g., pay-per-consultation, subscription-based, or free with premium options), and service scope (e.g., general consultations vs. niche specialties) [11]. Dr. Chunyu primarily operates as a text-based platform while also supporting audio consultations. It adopts a marketplace model, which allows patients to select from a wide network of licensed healthcare providers based on reviews, ratings, and expertise. It provides consultations across a wide range of medical specialties and operates free of charge with optional premium features [12]. Dr. Chunyu’s combination of affordability, anonymity, and accessibility has established it as the largest OMC platform in China [13], serving 65 million users with 200,000 registered doctors and a database of 70 million consultation records [14]. To safeguard patient confidentiality, the platform anonymizes consultations by removing identifiable information except for age, while publicly sharing doctors’ credentials, such as their position and affiliated institution.

Patient satisfaction refers to an individual’s subjective assessment of healthcare quality [15]. In China, satisfaction levels are notably low. Only 15.4% of patients report being satisfied with their doctors, a stark contrast to developed countries such as the United States, where satisfaction rates are significantly higher [16]. Patient-centered communication (PCC) is one approach to improving patient satisfaction. Nowadays, PCC is a fundamental value in medical education, prioritizing rapport, respect, empathy, and understanding over the more impersonal and detached nature of doctor–centered communication [17]. Research has shown that PCC in Chinese medical settings leads to positive outcomes, including improved patient adherence to medical instructions, enhanced emotional well-being, and stronger doctor–patient relationships [18,19]. PCC practices include delivering respectful and responsive care to patient’s values and preferences and encouraging them to actively participate in health decisions and share their perspectives on their health and healthcare [20]. Although PCC has been widely studied in previous studies in the context of in-person medical visits, less is known about how it manifests in sensitive topics within OMC settings. Also, Street (2017) argued that PCC should be understood as an interactive process rather than a one-directional form of communication from doctors. Within this framework, our research question is:

**RQ1:** How do PCC practices in OMCs addressing sensitive health topics contribute to enhancing patient satisfaction?

In addition to understanding the conversational themes that emerge in patient–doctor interactions [21], previous studies emphasized the need to examine communication strategies and discourse features in OMC settings from a more micro, linguistic perspective, especially given the prominence of language and the absence of non-verbal cues in online environments. For example, some studies analyzed how doctors use mitigating language, empathic responses, and authoritative discourse in OMCs from a linguistic lens [22,23,24]. Moreover, politeness theory provides an appropriate footing point for examining the linguistic structures of OMCs. By viewing communication as a rational, socially cooperative act, this theory illuminates how specific language features foster shared understanding [25]. Thus, the politeness theory as a guiding framework would help us understand the influence of these micro-linguistic markers on patient satisfaction.

The healthcare profession has often been criticized for exerting professional dominance over patients [21]. To address this, doctors are encouraged to adopt a more patient-centered approach by actively “listening more and speaking less” [26]. Previous studies have identified the number of conversational turns as a key discourse feature that can indicate dominance in interactions [27]. In the context of PCC, when doctors take fewer conversational turns than patients during OMCs, it could reflect a more patient-centered practice in OMCs. Therefore, we proposed the following hypothesis:

**H1.**  *The fewer conversational turns doctors take compared to patients, the higher the patient satisfaction in OMCs.*

Additionally, the use of polite language by doctors could empower patients, making them the focal point of medical consultations, which aligns with PCC practices [28]. Polite language fosters positive moral emotions, whereas impoliteness often triggers feelings of anger [29]. In Chinese culture, where politeness holds significant value, enhancing politeness in doctor–patient interactions has promoted more respectful exchanges and improved patient satisfaction [22]. Politeness markers are linguistic structures and expressions used to convey consideration and respect and soften or mitigate the force of certain speech acts [30]. As an umbrella term, politeness markers encompass a range of pragmatic devices that signal politeness in communication. Various taxonomies of politeness markers have been identified in different contexts. Commonly used politeness markers are ***polite words***, including phrases that express honor or request cooperation, such as “Please (请)”, as well as ***downtoner*** that aim to soften the force and imposition of the speaker’s remarks, such as “just (仅仅)” or “possibly (可能)” [30]. Previous studies have found that ***emojis*** can help convey politeness in online communication, especially for those with positive meanings [31]. Also, East Asians, in particular, tend to use emojis more frequently to align with situational contexts, in line with cultural norms that emphasize politeness and indirect communication [32]. In the context of OMCs, the ***expression of best wishes*** often serves as one of the politeness markers, such as “wish you all the best (希望一切都好)” [23]. Furthermore, the ***sentence-final particles*** (e.g., “ne (呢)”, “ya (呀)”) are often used as politeness markers in Mandarin Chinese to convey a friendly tone and reduce the perception of assertiveness or manipulation [33]. Yet, there is still a lack of empirical evidence identifying which specific politeness markers doctors use to influence patient satisfaction, particularly in the context of OMCs. To address this gap, we proposed the following hypothesis to explore the relationship between various politeness markers and patient satisfaction:

**H2.**  *The use of (a) polite words, (b) downtoners, (c) emojis, (d) expressions of best wishes, and (e) sentence-final particles by doctors is positively associated with patient satisfaction in OMCs.*

Previous research demonstrated that patients’ gender influences their satisfaction with doctor–patient communication. While both male and female patients create similar opportunities for doctors to demonstrate empathy, female patients exhibit greater emotional intensity, explicitly name their emotions, and display more intense emotional expressions than their male counterparts [34]. Sociolinguistic research also indicates that women are more likely than men to use polite language in their speech [35]. Current evidence might point out that female patients may have a greater need for politeness in medical interactions. Still, it remains unclear whether medical specialty (gynecology or andrology) influences patient satisfaction. We hypothesize that patients treated in different specialties may experience politeness markers differently. Thus, we aim to explore how key politeness markers (i.e., those with a statistically significant main effect in H2) interact with specialty types to influence patient satisfaction in OMCs based on the results of H2. Thus, we proposed:

**H3.**  *The effect of the key politeness markers on patient satisfaction will vary by medical specialty (gynecology vs. andrology), with the positive relationship between politeness and patient satisfaction being stronger for gynecology patients than andrology patients.*

Research on OMCs in the Chinese context has primarily employed qualitative methods to examine PCC practices (e.g., [23]). While these studies offer valuable insights into communication strategies, there remains a need for more comprehensive empirical evidence that examines both the thematic and linguistic features of patient–doctor interactions from both macro- and micro-level perspectives. This study aimed to advance current research by employing a mixed-methods approach to examine how PCC practices, including conversational themes and politeness markers, were applied in OMC settings, particularly in discussions of sensitive topics. The findings will contribute valuable resources for developing future guidelines to support doctors in their training and PCC practices, particularly in consultations addressing sensitive issues.

## 2. Materials and Methods

### 2.1. Data Collection

We collected conversations from publicly accessible cases, focusing on consultations in the gynecology and andrology specialties. The data were collected from 19 March to 22 March 2022, and included consultations between December 2018 and January 2021. Specifically, we gathered 149 conversations from gynecology specialty and 179 from andrology, resulting in a total of 328 cases. All consultations were primarily text-based, with some featuring additional audio responses from doctors. The audio responses were not included in the analysis. Since these data are openly available, no ethics review was required in mainland China. All sensitive and identifiable information related to doctors was removed to ensure privacy.

### 2.2. Study Design

First, we conducted a theme-oriented discourse analysis (TODA) to examine how PCC practices in sensitive health topics contribute to patient satisfaction. Following this, we conducted a content analysis of the collected consultations, coding each for the presence or absence of politeness markers and indicators of patient satisfaction. This two-step approach provided qualitative and quantitative insights into the role of PCC and the use of politeness markers in doctor–patient interactions.

### 2.3. Unit of Analysis and Measures

Each fully completed conversation was treated as the unit of analysis. The politeness coding scheme was adapted from the model of politeness markers [36], with additional politeness markers incorporated to better align with the Mandarin Chinese context. All politeness markers were coded as “1” if the doctor’s language included these markers in the consultation; otherwise, they were coded as “0”.

***Polite words***. Polite words refer to expressions that convey respect, request cooperation, or show consideration for the listener. Common examples include phrases such as “Please (请)”, “If you wouldn’t mind (如果您不介意)”, and “Dear (亲)”. These markers soften requests or commands, demonstrating politeness and respect in communication.

***Downtoners***. Downtoners are linguistic elements used to lessen the intensity or imposition of the speaker’s remarks. Examples include words like “just (仅仅)”, “possibly (可能)”, “maybe (也许)”, and “kind of (有点)”. These downtoners help moderate the tone of communication, making statements feel less direct or assertive, thereby creating a more polite and considerate interaction.

***Sentence-final particles***. Sentence-final particles are used at the end of sentences to express the speaker’s attitude, tone, or mood without altering the core meaning [33]. In Mandarin Chinese, common examples include “la (啦)”, “ba (吧)”, “ya (呀)”, and “ne (呢)”. These particles also help soften the tone, making the communication appear more polite and considerate toward the patient.

***Emojis***. Emojis are small digital icons used to express emotions, ideas, or concepts in electronic communication [37]. Researchers coded emojis used by doctors that conveyed positive meanings, such as 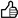, 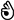, and 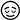. Given their widespread use in online interactions, emojis were included in the analysis as they can enhance the politeness of a message by visually conveying empathy, confirmation, or other positive emotions.

***Expressions of best wishes***. The study also considered expressions of best wishes at the end of conversations as one of the politeness markers [23]. In the Chinese context, these phrases typically focus on health, recovery, and well-being, such as “Wishing you a speedy recovery (祝您早日康复)” or “May you have a healthy body (祝您身体健康)”. These expressions demonstrate politeness by showing care and concern for the patient’s condition, helping to foster a more empathetic and supportive interaction.

***The number of doctor’s conversational turns***. Coders recorded the number of turns taken by both doctors and patients in each conversation. If the doctor took fewer turns than the patient, the consultation was coded as “1”; otherwise, it was coded as “0”. This metric provided insight into the interactional dynamics and served as an indicator of PCC practices.

***Patient satisfaction***. Consultations were coded as “1” if patients explicitly expressed gratitude or indicated that they accepted the doctor’s advice or considered the advice helpful. Conversely, if patients criticized the doctor, did not express appreciation, or indicate dissatisfaction, the consultation was coded as “0”.

### 2.4. Data Analysis

A theme-oriented discourse analysis (TODA) was conducted to answer the research question. TODA is a qualitative approach that examines how meanings are constructed and negotiated through language use within specific contexts [38]. TODA focuses on two dimensions of analysis: focal themes and analytic themes [39]. Focal themes pertain to key issues relevant to professional practices, such as patient-centered care, shared decision-making, the negotiation of medical knowledge, and rapport building. Analytic themes, informed by sociological and linguistic studies, explore discourse strategies such as face-work (i.e., behaviors aimed at managing one’s own image), contextualization cues, and footing (i.e., the alignment or stance individuals take during an interaction) [40], which help reveal the underlying dynamics of communication. Researchers acknowledge that the text-based nature can limit the presence of contextualization cues, making it difficult to fully explore certain analytic themes. As a result, this study focused on analyzing focal themes to gain insights into PCC practices. Two researchers independently reviewed and coded the texts using TODA as the analytical framework to identify meaningful focal themes aligned with PCC practices. Any discrepancies in coding were resolved through several rounds of discussion to ensure consensus.

**Figure 1:**
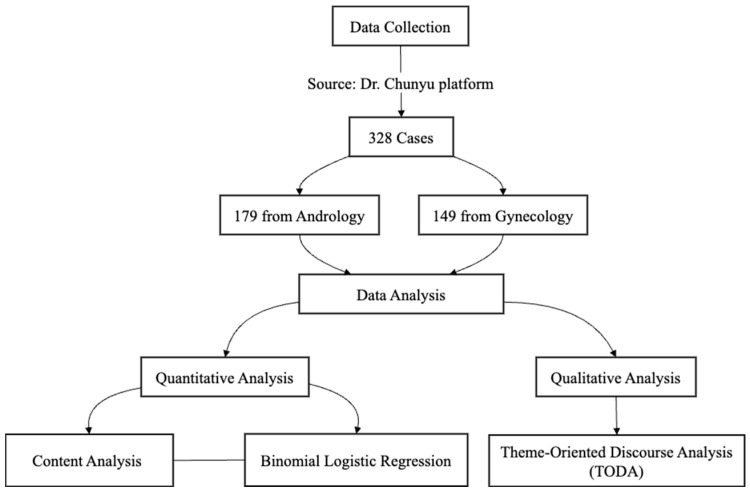
Flowchart illustrating the study procedure in the current research.

In addition to the discourse analysis, two undergraduate students were trained as coders for this study. To ensure coding reliability, 40 conversations (approximately 12% of the overall sample) were randomly selected from the original data pool for reliability testing. The two coders achieved high inter-coder reliability across all coding categories (Krippendorf’s *α*s= [0.90–1.0]). Binomial logistic regression was then conducted to assess the association between politeness markers and patient satisfaction, controlling for the patient’s age and the medical specialty areas. All analyses were conducted using the R *glm* package [41]. Figure 1 provides a detailed flowchart illustrating the key steps of our research design, including both qualitative and quantitative approaches to data collection and analysis.

## 3. Results

### 3.1. Enhancing Patient-Centered Communication in Sensitive Topics

Unlike the difficulties and ambiguity often encountered when discussing personal sexual matters during in-person clinical visits [42], the OMC setting provides a more comfortable environment for patients to seek medical advice. To address RQ1, our TODA revealed two focal themes within the data that suggested patient satisfaction. PCC practices, particularly normalization and collaborative decision-making, were essential in bridging the gap between patients and doctors in this context.

#### 3.1.1. Normalizing Sexual and Reproductive Health Issues

In OMCs, normalizing helps doctors convey understanding, validate patients’ concerns, and reduce stigma. The first step in effective OMCs is to establish trust and meet the patient’s emotional needs. For example, when a patient expressed pain and anxiety, the doctor responded with reassurance and a commitment to further investigation. By acknowledging the patient’s specific concerns and framing the diagnostic process as a collaborative effort, the doctor demonstrated empathy and care. This approach not only validated the patient’s feelings but also fostered trust in the doctor–patient relationship.

**Extract 1:**

Doctor: “I understand your worries about the recurring pain, and it’s important that we explore this together.”

Although nonverbal cues, such as body language or facial expressions, were absent in OMCs, doctors could still use comforting language to bridge this gap. Normalizing and legitimizing patients’ health concerns was providing emotional support to patients, a key component of PCC practices.

**Extract 2:**

Doctor: “Prostatitis is a common condition affecting nine out of ten adult men. It is a benign condition that does not impact life expectancy, so there’s no need to worry too much. When symptoms become noticeable, proper treatment should be administered, but if there are no obvious symptoms, it doesn’t require much attention. Managing this condition is 30% treatment and 70% lifestyle. Please pay special attention to your daily habits. It is crucial.”

In Extract 2, the doctor began by stating that prostatitis was a common condition, citing a statistic to normalize the issue and alleviate the patient’s concerns. By framing prostatitis as something nearly universal among adult males, the doctor effectively minimized potential anxiety and reassured the patient. Normalization empowered patients to engage more openly in discussions about their health, reducing the stigma or embarrassment associated with these issues. The doctor further employed a politeness strategy by avoiding overly prescriptive language. Instead of issuing direct instructions, the doctor offers suggestions, reducing the patient’s anxiety. The indirect and polite approach demonstrated an awareness of the patient’s emotional state, creating a communicative environment that fosters trust and understanding.

Another example highlighted the patient’s anxiety and feelings of stigma stemming from health information obtained from other sources.

**Extract 3**:

Patient: “I don’t know why I’ve had trichomoniasis, candidiasis, and bacterial vaginitis. I haven’t had unclean sexual activities, and I’ve always been careful about cleaning my genital area. So why do I keep getting these infections, doctor? I’ve been pretty careful in my daily life. Every time I search on Baidu (a search engine), it says it’s caused by an unclean sexual life.”

Doctor: “I’m not sure. It’s like catching a cold—sometimes I’m the only one who gets it, even when no one else around me is sick.”

Patient: “So, these vaginal infections aren’t necessarily caused by sexual activity, right? It feels quite embarrassing.”

Doctor: “There’s no need to feel embarrassed—there’s nothing shameful about being sick.”

The doctor responded by immediately normalizing the situation, framing the infection as a common occurrence, similar to catching a cold. This approach not only alleviated the patient’s anxiety but also reframed the condition in a non-stigmatized context, effectively reducing the shame often associated with sexual health concerns. The doctor reframed the infection as routine and manageable, thereby destigmatizing the issue and creating a non-stigmatizing environment. This empowered the patient to engage in the conversation more openly, exemplifying a patient-centered approach that encourages positive attitudes toward her condition. Such an approach could also enhance patients’ self-efficacy in managing sexual health conditions.

Moreover, by normalizing this condition, the doctor exercised epistemic authority, validating the patient’s experience and dispelling misconceptions. This validation was crucial, as it allowed the patient to address their health concerns without the burden of embarrassment or self-blame. This normalization strategy aligned with broader PCC practices in medical consultations, where healthcare professionals balance expertise with reassurance to foster patient participation and comfort. By blending empathy with medical knowledge, the doctor ensured the patient felt supported, creating a more inclusive and productive dialog around their health condition.

#### 3.1.2. Collaborative Decision-Making in Sensitive Health Issues

In our data corpus, tracking decision-making outcomes was challenging, as patients’ offline behaviors were not captured. However, we can still summarize the process by which patients and doctors reach agreements on health decisions. Unlike traditional face-to-face consultations, where the doctor primarily directs the conversation, the interactions observed in OMCs demonstrate a more participatory role for patients, encouraging shared decision-making. Extract 4 illustrated a case where a patient in a gynecological consultation was advised to undergo multiple examinations and expressed concerns about the necessity and frequency of these tests.

**Extract 4:**

Patient: “They gave me a lot of tests but didn’t prescribe any medication. After the abdominal ultrasound, they asked me to do a hormone panel, and after that, they asked for a thyroid function test. I asked to do a routine vaginal secretion test, but they wouldn’t let me.”

Doctor: “There’s nothing wrong with that. They’re trying to figure out why your endometrial lining is thickened.”

Patient: “So, should I go back and redo the tests next time I go to the hospital?”

Doctor: “A pelvic ultrasound is still necessary.”

Patient: “Hmm, what should I do in the meantime? How do I relieve this? I probably can’t go to the hospital for several days. Also, my period is about to start.”

Doctor: “You can use Bai’an cleansing solution and take Fuyan Kangfu tablets. After your period, you should get a pelvic ultrasound again to check the thickness of the endometrium. If the endometrial lining is within the normal range, there’s no need for a thyroid function test.”

Patient: “Okay. Will do.”

In this example, the patient actively engaged with the doctor, seeking clarification and negotiating the next steps in her care. The doctor’s response represented an effort to explain the necessity of the tests and involve the patient in the diagnostic reasoning process. This exchange highlighted a shift toward a more balanced, collaborative interaction between patient and doctor. The patient’s follow-up questions further illustrated this shift, as she took an active role in shaping the course of her care. The dialog also revealed a “decision tree” in the doctor’s discourse, outlining “what-if” scenarios and providing the patient with more opportunities to participate in the decision-making process.

This type of collaborative reassurance not only encouraged the patient but also reinforced the idea that healthcare was a joint effort. By framing medical consultations as partnerships, doctors could alleviate patient anxiety while promoting active participation in health management.

In cases where patients expressed reservations about treatment options, the doctor’s response became crucial. For instance, when a patient expressed hesitation about a suggested treatment, the doctor replied,

**Extract 5:**

Doctor: “I suggest you add medications that promote complete drainage and treat prostatitis. You could consider taking Flavonoid Piperazine Capsules and Tamsulosin Hydrochloride Capsules.”

Patient: “As long as I don’t get HIV, that’s all I care about. This is what worries me the most.”

Doctor: “Hmm, generally speaking, procedures like catheter treatment won’t lead to HIV infection. Any other questions?”

Patient: “I’ve been taking other Capsules for a month for my prostate, but I don’t feel any changes.”

Doctor: “If that’s the case, I recommend trying Flavonoid Piperazine Capsules and Tamsulosin Hydrochloride Capsules and see how it goes.”

Doctor: “If it still doesn’t work, combining traditional Chinese and Western medicine may be necessary.”

By offering alternative treatment plans and explaining the rationale behind each, the doctor not only addressed the patient’s concerns but also empowered the patient to make informed decisions about their health. The doctor introduced another decision pathway, using “if” scenarios to guide the patient through potential options.

### 3.2. Politeness Markers and Patient Satisfaction

The descriptive results of the coded variables are presented in Table 1. The distribution of politeness markers across the two medical specialties shows some variation. Specifically, gynecology doctors had more satisfied patients than the andrology doctors ($N_{Gynecology}$ = 81, 54.36%; $N_{Andrology}$ = 81, 45.25%). Apart from using polite words ($N_{Gynecology}$ = 67, $N_{Andrology}$ = 104) and emojis ($N_{Gynecology}$ = 9, $N_{Andrology}$ = 34), gynecology doctors exhibited a higher percentage of other politeness markers in their communication with patients.

Table 2 presents the zero-order correlations, revealing that polite words and expression of best wishes significantly correlate positively with patient satisfaction.

As shown in Table 3, regarding H1, doctors taking fewer conversational turns did not significantly influence patient satisfaction, as indicated by an odds ratio (OR) of 1.01 (*p* = 0.98). Thus, H1 was not supported. H2 (a)–(e) aimed to examine the relationships between different types of politeness markers and patient satisfaction. H2(a) and H2(d) were supported. The use of polite words (OR = 10.44, *p* < 0.001) and expressions of best wishes (OR = 3.56, *p* < 0.05) by doctors is significantly associated with an increased likelihood of patient satisfaction in OMCs. However, none of the other politeness markers, such as the downtoners (OR = 0.75, *p* = 0.34), the sentence-final particles (OR = 0.97, *p* = 0.93), or the emojis (OR = 0.95, *p* = 0.90), had a significant influence on patient satisfaction.

To assess whether the effects of polite words and expressions of best wishes vary by medical specialty (gynecology vs. andrology), we included interaction terms between these politeness markers and specialty in the regressions (H3). The findings indicated that neither the interaction between specialty area and polite words (OR = 0.38, *p* = 0.10) nor the interaction with expressions of best wishes (OR = 2.55, *p* = 0.36) was statistically significant, suggesting that the impact of these two politeness markers on patient satisfaction did not differ significantly between specialties.

## 4. Discussion

This study employed a mixed-methods approach to understand how patient-centered communication (PCC) practices and politeness markers influence patient satisfaction in discussions about sensitive health topics on a Chinese OMC platform. Through TODA, two key themes were identified within sensitive health discussions: normalizing sensitive health concerns and promoting collaborative decision-making in sexual and reproductive health discussions. In addition to the TODA results, quantitative results showed that the use of polite words and expressions of best wishes are positively associated with patient satisfaction. The interaction between the medical specialty area and both polite words and expressions of best wishes was non-significant, indicating that these politeness markers had a consistent impact on patient satisfaction across specialties. Other markers, such as downtoners and emojis, did not yield significant effects. Moreover, gynecological consultations exhibited higher patient satisfaction rates compared to andrology consultations. The zero-order correlation analysis revealed a positive association between age and the use of polite words and expressions of best wishes. This suggests that older patients may prefer more polite and respectful language, potentially reflecting generational differences in communication norms in China [43]. Additionally, fewer conversational turns by doctors were negatively associated with age, indicating that older patients tend to require more interaction and conversational exchanges during their consultations.

This study provides valuable quantitative evidence on the impact of politeness markers on patient satisfaction in OMCs. While previous studies have highlighted the importance of overall politeness in building trust and alleviating tension in OMCs, the focus has predominantly been on qualitative insights [9,44], leaving the quantitative effects of specific politeness markers largely underexplored. By addressing this gap, the current study demonstrates that the use of politeness markers significantly enhances patient satisfaction in OMC interactions. The coding scheme also contributes to the theoretical understanding of politeness theory in digital healthcare contexts, showing that conversational politeness strategies remain both applicable and impactful in computer-mediated medical communication. Additionally, this research incorporates localized politeness markers specific to Mandarin Chinese into the existing taxonomy of politeness markers, broadening the framework within non-Western contexts. The findings indicate that while politeness markers from Western frameworks (i.e., polite words) are effective in the Chinese OMC context, unique politeness markers are also emerging (i.e., expressions of best wishes). This cross-cultural dimension provides further empirical support for the universal relevance of politeness principles in various communication modalities.

While the role of politeness in fostering effective communication has been well-documented in face-to-face settings [45,46], its impact in text-based OMCs, where nonverbal cues like voice pitch, eye contact, and gestures are absent, remains underexplored. In these settings, conveying meanings relies heavily on verbal cues. Our study addresses this gap by revealing the effects of different politeness markers in OMCs. For instance, expressions of best wishes, typically associated with formal communication formats like letters or emails, were found to significantly enhance patient satisfaction in OMCs. In contrast, emojis, which can simulate nonverbal cues like facial expressions, showed no significant impact on patient satisfaction in this context. These findings deepen our understanding of communication in digital healthcare and offer practical guidance on the most effective politeness strategies. This knowledge can inform future research and best practices to improve patient-provider interactions in text-based OMC environments.

From the perspective of the PCC framework, our findings also extend the understanding of PCC by demonstrating that effective practices in offline settings, such as the linguistic choices of using polite expressions, are also valuable in OMCs. This study broadens the explanatory scope of PCC by showing that OMCs can provide an accessible online platform to normalize health concerns, fostering open discussions on sensitive topics and encouraging individuals to seek help. Through collaborative decision-making, OMCs facilitate personalized care, as well as serve as a resource for health education that may be unavailable through traditional educational channels [1]. By engaging patients in reasoning around treatments and prescriptions, doctors encourage active participation in sexual health management, breaking the silence and dispelling taboos that often surround these conversations. Our study emphasized the PCC in OMCs is particularly valuable in China, where sexual health issues are heavily stigmatized [47,48], and individuals often feel hesitant to address these topics in face-to-face consultations [42].

Furthermore, our findings highlight the importance of micro-level linguistic choices in digital healthcare settings, challenging the common notion that digital communication inherently leads to depersonalization since personalization might elicit individual reactance [49]. While prior PCC studies have generally focused on broader communication strategies, our research emphasizes that subtle linguistic elements, such as politeness markers, significantly shape patient experience and satisfaction. These linguistic choices convey empathy and attentiveness, helping to sustain a personal connection between doctor and patient within a digital environment [22]. By spotlighting these micro-level language nuances, our study advances PCC theory by demonstrating that specific word choices are critical in maintaining patient-centeredness, suggesting that digital healthcare platforms can effectively support relational communication even without face-to-face interaction.

Practically, our findings highlight the Internet’s potential as an accessible platform for patients to comfortably and openly discuss sensitive topics, such as sexual and reproductive health, with healthcare providers. With the rise in telemedicine, especially post-pandemic [50], our study offers a foundation for developing communication guidelines that enhance patient satisfaction in online medical settings. Specifically, incorporating polite words and expressions of best wishes could be effective strategies, as these elements function as affection-oriented language in Chinese OMCs, fostering positive interactions [9]. Our quantitative findings validate previous discourse studies, confirming that such language choices significantly impact patient satisfaction. These findings also suggest that patients in OMCs may seek additional emotional support, and these insights could help inform better practices for doctors engaging in online consultations, ensuring that telemedicine remains a patient-centered and empathetic form of care.

Although Dr. Chunyu, the OMC platform examined in this study, provides rankings of doctors based on rating scores and quantitative comments from patients [39], overall patient satisfaction remains below 50% in our data corpus. Interestingly, female patients report a 9.11% higher satisfaction rate than male patients. This difference may reflect broader societal expectations in China, where women often face social pressures to exhibit greater compliance and satisfaction, potentially influencing their responses more than those of men. Prior research has shown that men frequently approach evaluations from others with a sense of competition and confidence, while women typically show more receptivity to others’ opinions [51]. In the conversation corpus, we also observed a pattern where male patients frequently questioned physician competence and authority before fully engaging in the medical consultations, while female patients tended to view doctors’ suggestions as valuable insights into their health status. Consequently, women may be more inclined than men to incorporate doctors’ suggestions into their decision-making process. These findings suggest that gender may influence how patients perceive and respond to medical advice.

While this study provides valuable insights into the role of politeness markers and PCC in OMCs, it has limitations. The reliance on text-based consultations means that specific non-verbal cues, such as tone or body language, were not captured, which may have impacted the patient’s overall experience. Future research could explore video-based OMCs to examine how non-verbal communication influences patient satisfaction in digital consultations. Additionally, this study focused on gynecology and andrology consultations, which are inherently sensitive topics. Future studies could investigate whether these findings are generalizable to other medical specialties and whether the observed effects of politeness markers and PCC hold in less sensitive health contexts.

## 5. Conclusions

This study applied a mixed-methods approach to examine PCC practices and the impact of politeness markers on patient satisfaction in OMCs, focusing on sensitive topics. Through thematic and linguistic analyses, the current research identified two key conversational themes and specific politeness strategies that contribute to improved patient satisfaction. These findings emphasize the crucial role of PCC and politeness markers in enhancing patient experiences, particularly in sensitive health discussions within OMCs.

## Figures and Tables

**Table 1:** Descriptive results of coded variables.

	Gynecology N (%)	Andrology N (%)	Total N (%) ^2^
Fewer turns ^1^	80 (53.69%)	86 (48.04%)	166 (50.61%)
Polite words	67 (44.97%)	104 (58.10%)	171 (52.13%)
Downtoners	48 (32.21%)	41 (22.91%)	89 (27.13%)
Sentence-final particles	24 (16.11%)	24 (13.41%)	48 (14.63%)
Emojis	9 (6.04%)	34 (18.99%)	43 (13.11%)
Expressions of best wishes	19 (12.75%)	20 (11.17%)	39 (11.89%)
Patient satisfaction	81 (54.36%)	81 (45.25%)	162 (49.39%)

^1^ “Fewer turns” indicates doctors took fewer turns than patients. ^2^ The total number of consultations in the Andrology department was 179, while the Gynecology department had 149 consultations.

**Table 2:** Zero-order correlations of study variables.

	1	2	3	4	5	6	7	8	9
1. Fewer turns ^1^	-								
2. Polite words	−0.09	-							
3. Downtoners	0.00	0.06	-						
4. Sentence-final particles	−0.09	0.09	0.12 *	-					
5. Emojis	−0.01	0.06	0.03	−0.03	-				
6. Expressions of best wishes	−0.09	0.18 ***	0.11 *	0.03	0.11 *	-			
7. Medical specialty	0.06	−0.13 *	0.01	0.04	−0.19	0.02	-		
8. Age	−0.16 **	0.12 *	-0.04	−0.04	−0.09	0.15 **	−0.13 **	-	
9. Patient satisfaction	−0.05	0.49 ***	0.01	0.04	0.03	0.22 ***	0.09	0.06	-

^1^ “Fewer turns” indicates doctors took fewer turns than patients. * *p* < 0.05, ** *p* < 0.01, *** *p* < 0.001.

**Table 3:** The effect of politeness markers on patient satisfaction.

DV = Patient Satisfaction	Main Effect		Interaction		Interaction	
	OR	*p*	OR	*p*	OR	*p*
Fewer turns ^1 2^	1.01	0.98	1.01	0.98	0.10	0.99
Polite words ^1^	10.44	0.00 ***	17.26	0.00 ***	10.44	0.00 ***
Downtoners ^1^	0.75	0.34	0.74	0.32	0.76	0.37
Sentence-final particles ^1^	0.97	0.93	0.96	0.92	0.94	0.87
Emojis ^1^	0.95	0.90	0.95	0.90	0.93	0.86
Expressions of best wishes ^1^	3.56	0.01 **	3.66	0.01 **	2.52	0.12
Interactions						
Specialty * Polite words			0.38	0.10		
Specialty * Expressions of best wishes					2.55	0.36
Medical specialty (ref. = Andrology)	2.60	0.00 **	4.46	0.00 **	2.60	0.00 **
Age	1.00	0.86	1.00	0.83	1.00	0.89

^1^ All the independent variables were dummy coded variables, with no = 0 as the reference group. ^2^ “Fewer turns” indicates doctors took fewer turns than patients. * *p* < 0.05, ** *p* < 0.01, *** *p* < 0.001.

## Data Availability

The raw data presented in the study are openly available on the Dr. Chunyu platform. The organized data supporting this article’s conclusions will be made available by the authors upon reasonable request.
